# One Molecule for Mental Nourishment and More: Glucose Transporter Type 1—Biology and Deficiency Syndrome

**DOI:** 10.3390/biomedicines10061249

**Published:** 2022-05-26

**Authors:** Romana Vulturar, Adina Chiș, Sebastian Pintilie, Ilinca Maria Farcaș, Alina Botezatu, Cristian Cezar Login, Adela-Viviana Sitar-Taut, Olga Hilda Orasan, Adina Stan, Cecilia Lazea, Camelia Al-Khzouz, Monica Mager, Mihaela Adela Vințan, Simona Manole, Laura Damian

**Affiliations:** 1 Department of Molecular Sciences, “Iuliu Hațieganu” University of Medicine and Pharmacy, 400347 Cluj-Napoca, Romania; romanavulturar@gmail.com (R.V.); adinachis82@gmail.com (A.C.); 2 Cognitive Neuroscience Laboratory, Department of Psychology, Babes-Bolyai University, 400029 Cluj-Napoca, Romania; 3 Faculty of Medicine, “Iuliu Hațieganu” University of Medicine and Pharmacy, 400347 Cluj-Napoca, Romania; pintilie.sebastian.romeo@elearn.umfcluj.ro (S.P.); botezatu.alina@elearn.umfcluj.ro (A.B.); 4 Chemistry Department, Oxford University, Oxford OX1 3TA, UK; ilinca.farcas@new.ox.ac.uk; 5 Department of Physiology, “Iuliu Hațieganu” University of Medicine and Pharmacy, 400347 Cluj-Napoca, Romania; 6 Internal Medicine Department, 4th Medical Clinic, “Iuliu Hațieganu” University of Medicine and Pharmacy, 400347 Cluj-Napoca, Romania; adela.sitar@umfcluj.ro (A.-V.S.-T.); hilda.orasan@umfcluj.ro (O.H.O.); 7 Department of Neuroscience, “Iuliu Hațieganu” University of Medicine and Pharmacy, 400347 Cluj-Napoca, Romania; dora.stan@umfcluj.ro (A.S.); monica.mager@umfcluj.ro (M.M.); adelavintan@gmail.com (M.A.V.); 8 Department Mother and Child, “Iuliu Hațieganu” University of Medicine and Pharmacy, 400347 Cluj-Napoca, Romania; cecilialazea@umfcluj.ro (C.L.); calkhuzouz@umfcluj.ro (C.A.-K.); 9 Emergency Clinical Hospital for Children, 400394 Cluj-Napoca, Romania; 10 Department of Radiology and Medical Imaging, “Iuliu Hațieganu” University of Medicine and Pharmacy, 400347 Cluj-Napoca, Romania; simona.manole@gmail.com; 11 Department of Radiology, “N. Stancioiu” Heart Institute, 400001 Cluj-Napoca, Romania; 12 Department of Rheumatology, Emergency Clinical County Hospital Cluj, Centre for Rare Autoimmune and Autoinflammatory Diseases (ERN-ReCONNET), 400347 Cluj-Napoca, Romania; ldamian.reumatologie@gmail.com; 13 CMI Reumatologie Dr. Damian, 400002 Cluj-Napoca, Romania

**Keywords:** Glut1, epilepsy, movement disorders, inborn errors of metabolism, cognitive impairment, glucose uptake, flow cytometry, ketogenic diet, *SLC2A1*, inflammation

## Abstract

Glucose transporter type 1 (Glut1) is the main transporter involved in the cellular uptake of glucose into many tissues, and is highly expressed in the brain and in erythrocytes. Glut1 deficiency syndrome is caused mainly by mutations of the *SLC2A1* gene, impairing passive glucose transport across the blood–brain barrier. All age groups, from infants to adults, may be affected, with age-specific symptoms. In its classic form, the syndrome presents as an early-onset drug-resistant metabolic epileptic encephalopathy with a complex movement disorder and developmental delay. In later-onset forms, complex motor disorder predominates, with dystonia, ataxia, chorea or spasticity, often triggered by fasting. Diagnosis is confirmed by hypoglycorrhachia (below 45 mg/dL) with normal blood glucose, 18F-fluorodeoxyglucose positron emission tomography, and genetic analysis showing pathogenic *SLC2A1* variants. There are also ongoing positive studies on erythrocytes’ Glut1 surface expression using flow cytometry. The standard treatment still consists of ketogenic therapies supplying ketones as alternative brain fuel. Anaplerotic substances may provide alternative energy sources. Understanding the complex interactions of Glut1 with other tissues, its signaling function for brain angiogenesis and gliosis, and the complex regulation of glucose transportation, including compensatory mechanisms in different tissues, will hopefully advance therapy. Ongoing research for future interventions is focusing on small molecules to restore Glut1, metabolic stimulation, and *SLC2A1* transfer strategies. Newborn screening, early identification and treatment could minimize the neurodevelopmental disease consequences. Furthermore, understanding Glut1 relative deficiency or inhibition in inflammation, neurodegenerative disorders, and viral infections including COVID-19 and other settings could provide clues for future therapeutic approaches.

## 1. Introduction

Glucose transporter type 1 (Glut1) is a trans-membrane protein which is responsible for the passive transport of D-glucose, D-galactose, D-glucosamine, and the glucose analogues 2-deoxy-D-glucose (2-DOG) and 3-O-methyl-D-glucose (3-OMG) [1]. Glut1 has a ubiquitous distribution, being highly expressed in the brain and in the erythrocytes, and is mainly responsible for the basal-level cellular uptake of glucose into many tissues [1]. Glut1 was the first identified member of the GLUT family carriers to provide basal glucose uptake across the blood–tissues barriers, including the blood–brain barrier (BBB) [2,3].

Glucose transporter type 1 deficiency syndrome (Glut1DS), first described in 1991 by Darryl De Vivo, is mainly an autosomal dominant inborn error of brain energy metabolism caused by impaired glucose transport into the brain [4]. Nowadays, the knowledge regarding this defect demands international consensus statements for diagnosis and treatment [5]. The disease is produced mainly by mutations in the *SLC2A1* gene encoding the Glut1. The defects are characterized by the impaired transport of glucose across the BBB, leading to low glucose levels in the cerebrospinal fluid (CSF), known as hypoglycorrhachia [6].

The membrane protein glucose transporters are part of one of the largest families of the transporters, named the Major Facilitator Super-family (MFS), a branch of the Sugar Porters (SP) sub-family, the members of which are responsible for the uptake of glucose and other monosaccharides or disaccharides [1]. An over-expression of *GLUT1* and other glucose transporters genes is observed for a wide variety of malignant cells [7].

Generally, the Glut family transporters are electroneutral, except for Glut9 (mainly electrogenic transporters for urates), Glut12 (voltage-dependent) and Glut13 (proton-coupled) [8]. Glut12, initially considered a Glut4-like transporter (involved in insulin-dependent glucose transport), actually has physico-chemical properties similar to Glut1, and is found in renal tubules, epithelial jejunal and pyloric glands, adipose tissue, the liver, and skeletal muscle, as well as in the thyroid, adrenal and pituitary glands [9,10] (see Table 1).

SWEETs, a newly added sugar transporter family for humans, may mediate both cellular uptake and efflux, and have low affinity for sugars; SWEETs are highly conserved [11].

**Figure 1:**
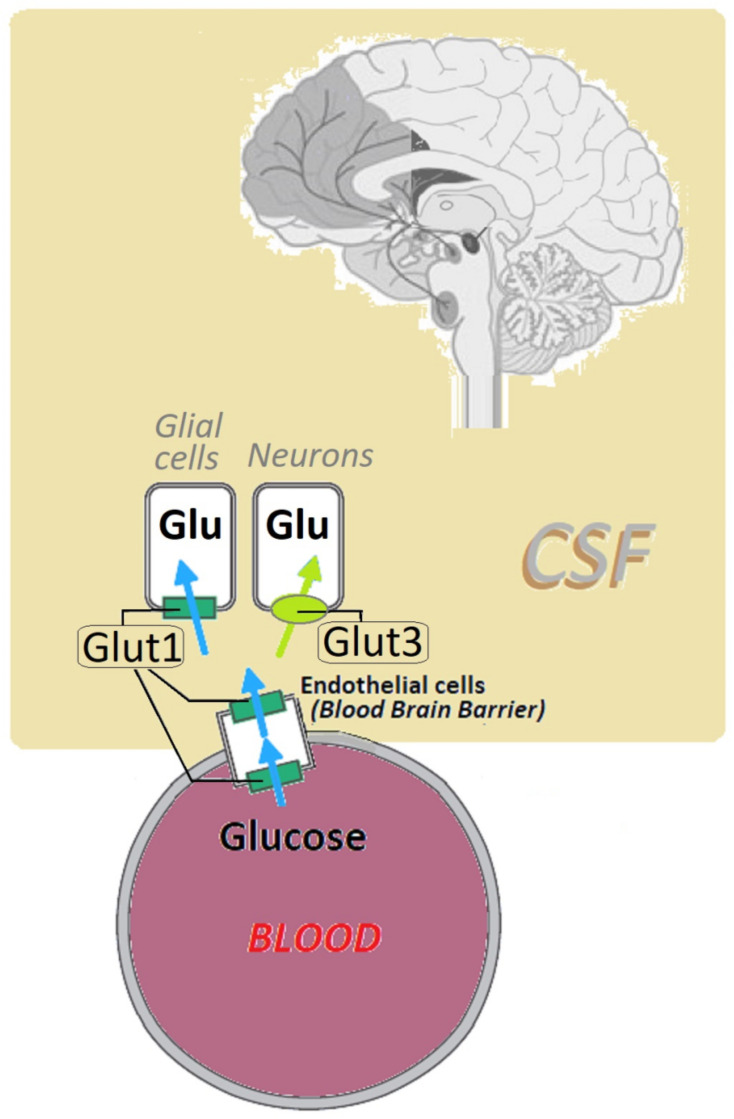
Representation of the main glucose transporters into the brain, Glut1 and Glut3, belonging to facilitated, “passive” transporters (encoded by members of the SLC2 gene family). Transport across cell membranes is depicted by arrows; localization and known defects of Glut1 are shown by green rectangular symbols (based on [12,15]). Legend: Glu, glucose; CSF, cerebrospinal fluid.

The Glut1 involvement in glucose transport for neuronal function is significant, as it regulates the blood supply and is the driving force for transport, depending on the D-glucose concentration gradient between blood and brain interstitium; see Figure 1 [15].

## 2. Glucose Transporter Protein Type 1 (Glut1): Structure and Function

**Figure 2:**
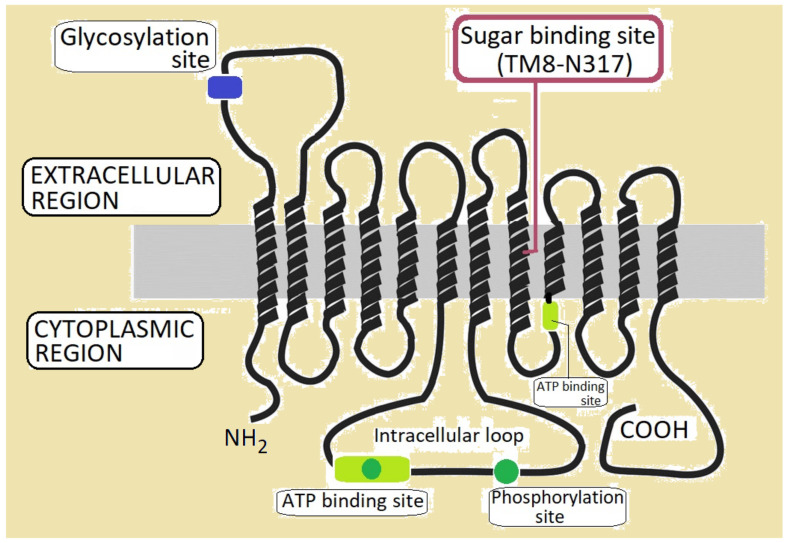
A model of the Glut1 structure with 12 TM domains, and the main functional sites: ATP binding sites, the phosphorylation site, and the sugar binding site in position N317 of the TM8 domain.

Glut1 is a glycoprotein which is present in most tissues but highly expressed in brain endothelial cells, glial cells, erythrocytes and placenta [4,16,17]. Along with the other 13 facilitative glucose transporters (Glut2-Glut14), Glut1 is encoded by solute-linked carrier family 2, with the subfamily A gene, member 1 (*SLC2A1* or *GLUT1*) mapped on chromosome 1 (region 1p34.2) [18]. Initially, it was purified from human erythrocyte membranes through sodium dodecyl sulphate-polyacrylamide gel electrophoresis (SDS-PAGE), as a protein of 55 kDa [19]. The first complete gene and amino acid sequence of the protein was obtained using a human HepG2 hepatoma cell line [2]. Glut1 is an integral membrane protein of 492 amino acids, with a conformational model comprising 12 trans-membrane segments (TM1-TM12) with the N- and C-domains located into the cytoplasm. Besides the TM domains, the Glut1 protein presents an intracellular loop (between TM6 and TM7) and a glycosylation site in the first extracellular loop (Figure 2). Likewise, toward the C-domain (TM7-TM12) the protein has several functional sites: ATP binding sites, phosphorylation sites, and the sugar binding site (in the TM8-N317) [3,18,20].

The main role of the N-domain (TM1-TM6) is the regulation of the protein conformation during sugar transport; the three-dimensional inward-facing computer model showed a pseudo-symmetry between the N-domain and the C-domain, supporting the model through which the Glut1 domains arose from a duplication of a protein with six transmembrane domains [18,19,21,22]. The expression of Glut1 is age-related [13]: (a) during embryonic development, the transporter is highly expressed in the proliferating cells; (b) after birth, during the first months of life, it is present mainly in the brain, myocardium and skeletal muscles; and (c) in the adult period, the main tissues rich in Glut1 are the brain and the erythrocytes. Dysfunctional mutations of this protein may lead to defective transport across the BBB, leading to hypoglycorrhachia, one of the main signs of Glut1DS. By contrast, the over-expression of *GLUT1* in tumor cells is a prognostic tool for cancer [20].

## 3. Pathophysiology

**Figure 3:**
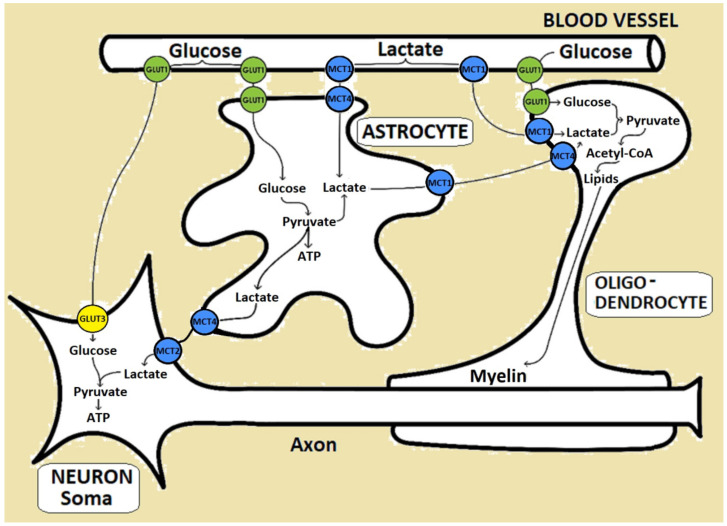
Representation of glucose and lactate fluxes through the BBB. Glucose transporters: Glut1 (depicted in green) is expressed in endothelial cells (part of BBB) and in glial cell membrane; Glut3 (yellow) is expressed in neuron membrane. MCT (monocarboxylate transporters family) is depicted in blue: different isoforms (MCT1, MCT2, MCT4) are expressed in endothelial cells (part of the BBB), neurons, astrocytes, and oligodendrocytes.

The Glut1 anomalies impair the glucose supply to glial cells and neurons (Figure 3), and Glut1DS patient observation did not provide information about any major disturbance during the placental transport of glucose [3,4]. The brain is one of the most active organs, receiving 20–25% of the total body glucose [17]. Furthermore, its glycogen storage capacity is very low, and it needs a continuous supply of glucose, or ketone bodies as an alternative energy source [16]. It is not clear if the impaired transport of the other substrates of Glut1 (other sugars than glucose) contributes to the pathophysiology of Glut1DS. In a mouse model, the pathology of the classical type of Glut1DS can be recognized early in life, particularly at the stage of brain angiogenesis [23].

In cell cultures, Glut1 deficiency decreases cellular ATP functions and activates phosphor-AMP kinase (AMPK), the cellular energy sensor, suppressing protein synthesis downstream and triggering cell-growth arrest factors p53 and Cdkn1a [24]; nevertheless, in Glut1DS animal models, AMPK was not increased and p53 was even decreased [24,25]. Besides *SLC2A1* (*GLUT1*) haploinsufficiency in Glut1DS and the roles of other glucose transporters, an important explanation resides in the role of Glut1 in the modulation of cerebral angiogenesis through the tip cells, brain dysfunction, and neuroinflammation leading to gliosis in Glut1DS [24,25]. The role of the neurotrophic factor BDNF, which is reduced in Glut1DS, is to be further clarified [24]. BDNF is also involved in angiogenesis, is one of the reasons for the selective brain dysfunction, and is conceivably related to microcephaly in Glut1DS [24].

The deficiency of Glut1 results in hypoglycorrhachia, low lactate levels, low brain energy metabolism, reactive astrocytosis, microcephalia, and several clinical signs such as epileptic episodes that are often unresponsive to anticonvulsants; in some cases, seizures can be aggravated by fasting [12]. A complex motor disorder becomes apparent with increasing age, with signs of spasticity, dystonia, ataxia and chorea. Global developmental delay becomes apparent in almost all patients with Glut1DS [7,12].

Glut1DS has a heterogeneous clinical picture with a classical phenotype (with early-onset epileptic encephalopathy and other cardinal features; see subchapter 4, clinical picture) and a non-classical phenotype with less-severe signs [12,26]. As previously suggested, beside the inadequate transport of glucose across the BBB, the reduced uptake into the astrocytes and oligodendrocytes may also contribute to the disease [27,28]. Due to the low concentration of glucose, limited brain glycogen storage and nucleic acid synthesis in the affected pentose phosphate shunt can cause metabolic stress. The pathway of D-glucose in the brain is clearly defined: from the brain capillaries, it is taken up by astrocytes which set up the metabolic path for glucose or its glycolytic metabolite, L-lactate, to neurons (Figure 3) [15]. Besides this, glucose can diffuse trough the gap junctions and follow the neuronal pathway directly. The neuronal activity also has implications in the interplay of D-glucose and L-lactate concentrations [15].

As an alternative source of energy for the brain, ketone bodies enter into the brain using a different pathway—by monocarboxylic transporter (MCT1) system—and provide an alternative source of acetyl-CoA under conditions that affect pyruvate synthesis from glucose [29]. A remarkable homology between humans’ and rodents’ Glut1 structures and localizations was noticed [4]. In animal Glut1DS models, mutant mice develop a significant embryopathy with various neurologic malformations, including microcephaly, anencephaly and anophthalmia [30]. Similar features were identified in humans, but affected infants appear normal at birth [29].

## 4. Clinical Picture of Glut1 Deficiency Syndrome

The classical form of Glut1DS presents as an early-onset (during the first year of life) encephalopathy with three cardinal features: severe epilepsy, a complex movement disorder, and developmental delay, including microcephaly. The predominant manifestations are seizures (that usually begin during the first year of life), after an uneventful fetal and neonatal development (due to immature tight junctions in the BBB that allow paracellular glucose transport) [12]. Five seizure types were described: generalized tonic or clonic, myoclonic, atypical absence, atonic, and unclassified [12,23,29,31,32,33,34]. Eye movements, an early warning sign which is sometimes inaugural for Glut1DS, usually disappear after early infancy [35]. The aberrant gaze saccades are peculiar head-eye movements, noticeable in one third of infant patients; they can be distinguished from opsoclonus by the presence of a clear inter-movement fixation interval and the association of a same-direction head movement [35,36,37]. In later childhood, they can be variable, and are often unresponsive to anticonvulsants drugs. The horizontal eye movements originate in the paramedian pontine reticular structure, while the vertical eye movements originate in the mesencephalic reticular formation [38]. However, according to the EEG tests, these episodic movements are non-epileptic. In neonatal patients, Glut1DS may evolve with cyanotic spells and atonic drop attacks [38]. Glut1DS responds for about 5% of myoclonic-astatic epilepsy and for 10% of early-onset absence-epilepsy cases, respectively [38]. Non-epileptic paroxysmal events with episodic ataxia, weakness, Parkinsonism or alternating hemiplegia may develop later in life, often triggered by fasting. The EEG during seizures shows multifocal spike-wave discharges in adults [38].

The non-classical type of the disease is milder, and consists mostly of only one or two of the cardinal features, e.g., isolated early-onset absence epilepsy or an isolated movement disorder without epilepsy [12]. This diagnostic should be considered in any unexplained seizures of various types in early age [39]. During childhood or adolescence there is a transition towards movement disorders [40]. Nevertheless, apneic episodes or abnormal episodic eye movements simulating opsoclonus may precede the onset of seizures by several months [23,29]. In the non-classical Glut1DS phenotype there are usually no seizures, but intellectual disability, impaired language development and intermittent ataxia are seen, sometimes with associated microcephaly [32,38]. Other common manifestations in the non-classical phenotype of the disease are spasticity, dyspraxia and paroxysmal exercise-induced dyskinesia (PED) with transient involuntary movements such as dystonia, chorea or ballism [32]. PED has been found to be an allelic variant of classic Glut1DS [12]. Clinically, these patients have a normal head circumference, normal inter-ictal neurologic examination, and a lesser decrease of CSF glucose concentration when compared with classic Glut1DS [12]. Manifestations with only minimal symptoms in adults have also been described [40].

## 5. Genetics and Metabolic Changes

Glut1DS is mainly caused by pathogenic variants of the *SLC2A1* gene; approximately 90% of patients exhibit a heterozygous de novo mutation, while 10% inherit a deficient gene from their parents (which seems to correlate with a milder phenotype) [29,32]. In most cases, Glut1DS exhibits an autosomal dominant inheritance pattern. However, there have been multiple reported cases of autosomal recessive mutations, for which heterozygous carriers are asymptomatic [5,29], indicating that the inheritance pattern depends on the mutation pathogenicity and the subsequent haploinsufficiency degree [41]. The birth incidence of Glut1DS has been estimated to be between 1:24,000 [42] and 1:90,000 (in Australia) [43], with a similar result in Denmark (1:83,000) [44]. To date, about 500 cases have been reported worldwide. Due to the fact that many neurological conditions can cause the same symptoms as in Glut1DS, the disorder may be under-diagnosed; some studies suggested that about 105,000 patients suffer from Glut1DS worldwide [24].

So far, over 200 types of genetic defects have been described, among which are missense, nonsense, frameshift, and splice-site mutations, and large fragment deletions [12,32]. Of these, missense mutations seem to be associated with a milder phenotype, but no definite genotype–phenotype correlation has been described [5,45]. Pathogenic variants in *SLC2A1* are most often identified by sequencing (81–89% of cases), and less often by deletion/duplication analysis (11–14%) [29]. An asymptomatic parent harboring the pathogenic variant implies a mosaic state [29].

Of note, allelic disorders with overlapping features include GLUT1 deficiency syndrome with pseudohyperkalemia and hemolysis, GLUT1 deficiency syndrome-2 (GLUT1DS2), dystonia-9 (DYT9), and idiopathic generalized epilepsy-12 (EIG12) (https://www.omim.org, accessed on 20 April 2022).

Different *SLC2A1* variants could destabilize Glut1 native interactions or generate novel interactions, initiate protein misfolding, enhance protein aggregation, or be influenced by non-coding RNA genes or by defects in translation, transcription, processing, and activating Glut1 protein [5,46]. In a small proportion of patients with Glut1DS, no *SLC2A1* genetic defect can be identified, even after the additional use of MLPA (multiplex ligation-dependent probe amplification) analysis to detect copy number variations [12]. For these patients, other factors that may lead to altered Glut1 function, such as changes in other related genes; downstream malfunctions in transcription, translation and protein folding and function; or improper regulating processes have been proposed [12]. For instance, a frameshift deletion in the *PURA* gene, coding for a transcriptional and translational regulator protein, led to hypoglycorrhachia, along with a lowered Glut1 expression on the membrane of peripheral red blood cells [47]. Sometimes the hallmarks of the clinical and biological picture may be given by other genes, coding for different membrane transporters such as SLC9A6, enzymes, receptors or transcriptional factors, or involving other mechanisms such as protein recycling [48]. In individual patients, mutations reported to modify the clinical picture in Glut1DS-involved genes include *SCN8A*, *ATP1A3*, *KCNQ2*, *NALCN*, *DNM1*, *MAN2B* and *UNC13A* [48].

## 6. Diagnosis

The diagnosis of Glut1DS is complicated by the phenotypical diversity and evolution with age of the disease [24,32]. An important laboratory investigation for Glut1DS is the low CSF:blood glucose ratio, with blood glucose being normal [29]. The CSF:blood glucose ratio should be measured in a metabolic steady-state, during non-ictal periods [12].

The CSF glucose is investigated by lumbar puncture after four–six hours of fasting. For the diagnosis of Glut1DS, the CSF glucose should be under 48.6 mg/dL (<2.7 mmol/L), but Glut1DS should be suspected in all children with CSF glucose concentration below 45 mg/dL (normal >59.4 mg/dL) [49]. In affected patients, the values vary considerably (range 16.2–52.2 mg/dL), being higher in milder phenotypes and in paroxysmal movement disorders [12].

The CSF to blood glucose ratio is normally 0.6 (0.65 ± 0.1) [49]. In the absence of a CNS infection or hypoglycemia, a CSF to blood glucose ratio value under 0.45–0.5 (range 0.19–0.52) is diagnostic for Glut1DS [12,49].

Typically, CSF lactate is low or normal, while the cell count and proteins are normal, which helps to differentiate between Glut1DS and other diseases (meningitis or encephalitis) in which lactate levels are high [32].

Genetic testing for mutations in *SLC2A1* is also recommended when Glut1DS is suspected, although a negative result cannot rule Glut1DS out [50]. Thus, sequencing or duplication/deletion analysis can be employed when clinical findings and/or hypoglycorrhachia are suggestive of Glut1DS.

Two other methods have been proposed to analyze the function of Glut1: (a) an erythrocyte 3-O-methyl-D-glucose (3-OMG) uptake assay, with a 74% uptake cutoff for abnormally low levels indicative of Glut1DS, and (b) a red blood cell Glut1 surface expression test using flow cytometry analysis in circulating erythrocytes, which is at least 20% lower in affected patients [12,51,52,53].

Other routine laboratory tests and inter-ictal EEGs are normal. If they are abnormal, an improvement in the EEG with glucose intake may be of diagnostic value (https://www.omim.org, accessed on 20 April 2022). Ictal EEGs may show epileptiform discharges in infants and a generalized spike-wave pattern in older children.

Regarding the structural brain changes, cerebral MRI may show in a fourth of patients delayed myelination, the hyperintensity of the subcortical U-fibers, and the enlargement of perivascular Virchow spaces [5]. Functional imaging techniques, such as 18F-deoxyglucose-positron emission tomography (^18^F-FDG-PET), may identify a decrease in cortical, cerebellar and thalamic glucose uptake, particularly in the mesial temporal lobe, with a relative signal increase in the basal ganglia and striatum, mainly in the caudate nucleus [5,12].

The differential diagnosis of Glut1DS includes, without being limited to, (a) other causes of neuroglycopenia such as chronic or intermittent hypoglycemia (e.g., familial hyperinsulinism); (b) opsoclonus-myoclonus syndrome; (c) all causes of neonatal seizures and acquired microcephaly, such as Rett syndrome (early presentations), Angelman syndrome, and infantile forms of neuronal ceroid-lipofuscinosis; (d) cryptogenic epileptic encephalopathies with developmental delays; (e) familial epilepsies with autosomal dominant transmission; (f) episodes of paroxysmal neurologic dysfunction which are responsive to carbohydrate intake, especially when manifesting as ataxia, cognitive dysfunction, alternating hemiparesis, or seizures; and (g) movement disorders including dystonia [29].

## 7. Treatment, Prognosis and Research

During fasting, ketone bodies provide an alternative fuel to the brain, and this metabolic state can be induced by a high-fat, low-carbohydrate diet called a ketogenic diet (KD), restoring the brain energy metabolism in Glut1DS, as ketone bodies’ transport at the BBB is not dependent on Glut1. The classic ketogenic diet (4:1 and 3:1 ratios of calories from lipids and non-fat sources, respectively) in Glut1DS patients may effectively control seizures and movement disorders, and improve development. Calcium and multivitamin supplements are necessary on a KD. In contrast to intractable childhood epilepsy, the KD in Glut1DS patients should be maintained throughout childhood and adolescence, until cerebral glucose requirements decrease. The early initiation of this therapy supports brain growth and normal brain function in the adult period of life; the disease generally stabilizes after puberty [12]. The main beneficial effect of the KD is the control of seizures; after initiating therapy, the patients generally have a rapid improvement (over days) in seizure control. Other positive effects of a KD are related to the improvements of motor (i.e., paroxysmal dyskinesias) and cognitive symptoms, but the results are variable [12,54]. The prolonged administration of the diet might lead to long-term adverse effects: hypercholesterolemia, growth impairment, acidosis, kidney stones, and a high risk of metabolic syndrome and cardiovascular disease in adults [55,56,57]. The KD may result in the loss of adherence, metabolic disturbances, and gut microbiome alterations. However, in the long-term the KD did not significantly change nutritional status, body fat, glucose and lipid profiles, nor ghrelin or leptin [58]. Nevertheless, after 3 months of KD, a significant increase in *Desulfovibrio* spp.—thought to be associated with colonic inflammation—was noted in Glut1DS patients, making an empirical trial of pro- or pre-biotics reasonable [59].

Furthermore, the administration of carnitine (deficient in KD, but with an important role in mitochondrial lipid oxidation) and α-lipoic acid facilitates glucose transport through Glut4, another transporter expressed in the brain [60,61].

In order to compensate for some of the adverse effects, the modified Atkins diet was proposed for adults, and remains a promising alternative by initiating an improvement in cognitive ability and epilepsy; it is less restrictive (any amounts of protein and fat might be consumed, but there is a limited carbohydrate intake of 10 g/day) [62]. However, despite adequate treatment, seizures and a variable degree of impairment may persist in several Glut1DS patients; paroxysmal events and the impairment of expressive language appear to be difficult to treat in adults [12]. Treatment decisions are less clear in atypical variants of Glut1DS, mainly in late-onset or in paucisymptomatic patients, due to limited experience [5].

Artificial ketones (ketoesters and triheptanoin—a synthetic medium-chain triglyceride) represent anaplerotic substances, and provide an alternative energy source, see Table 2; thus, they could represent a novel therapeutic option [63,64]. The use of dietary antioxidants, such as α-lipoic acid (thioctic acid) is under investigation [5,12].

Another proposed alternative in patients with compliance or intolerance problems is the modified high amylopectin cornstarch and low glycemic index diet, providing a steady-state glucose transport into the brain [65]. Research projects investigate families of medium chain triglyceride and hexose transporters that may provide metabolic fuel for the brain [5,63].

Diazoxide administration (a medication used to treat low blood sugar, interfering with insulin release through its action on potassium channels) was proposed to contribute to raising the blood and CSF glucose levels in a patient with Glut1DS who did not respond to a KD. This provides a higher level of glucose in CSF, and clinical improvement, being a reliable long-term treatment when associated with continuous glucose monitoring [66].

Drug administration in patients with Glut1DS remains an additional strategy to control seizures or paroxysmal dyskinesias (which, it seems, respond well to acetazolamide). Even if the symptoms are improved, the anti-epileptic therapy is unable to correct the brain energy necessary for growth and development [5,54].

Furthermore, the avoidance of Glut1 dietary or pharmacologic inhibition is important. In vitro studies showed that methylxanthines (caffeine, theophylline), tricyclic antidepressants, sodium valproate, barbiturates, diazepam, chloralhydrate, and ethanol are nonspecific inhibitors of Glut1 function [5,27,67].

Research on disease mechanisms has identified novel targets for therapy, focusing on (a) the molecular mechanisms involved in the metabolic defect (brain glucose depletion) and neurological consequences, (b) the type of transporter dysfunction, and (c) the imagistic investigations (PET-based investigation and MRI (Magnetic Resonance Imaging)) of human brain metabolism [5].

## 8. Glut-1 Deficiency in Other Tissues: Expanding the Clinical Phenotype?

While Glut1DS is primarily a brain disorder, Glut1 haploinsufficiency can also affect other organ systems relying on glucose to fuel their energy requirements [24]. A better understanding of the role of glucose transporters in various settings has revealed unexpected potential functions of Glut1 modulation, as tissue functions are partly controlled by metabolic substrates [68]. Glut1 is also expressed in the retina, ciliary muscle, peripheral nerve endoneurium and perineurium, placenta, and testis, etc. [69]. Rare features described in Glut1DS include periventricular calcifications, pseudohyperkaliemia, parkinsonism, writer’s cramp, nocturnal muscle cramps, cyclic vomiting, and others [5].

The proliferating cells have higher glucose requirements [68]. Warburg’s effect, i.e., oxidative glycolysis performed even in the presence of enough oxygen, increasing glucose and glutamine uptake, and favoring anabolism, was observed in rapid growth requirements such as embryonic development, wound healing, T cell activation or pluripotent cell proliferation [70].

### 8.1. Glut1 in Vessels

Besides the brain energy deprivation, the Glut1DS clinical picture also results from the brain vasculature insufficiency [24]. In endothelial cells, glycolysis promotes vessel branching and migration [68]. Endothelial tip cells, which are highly glycolytic and critical for brain angiogenesis, are significantly reduced in quantity and quality in Glut1DS [24]. Moreover, endothelial cell-specific Glut1 haploinsufficiency was involved in triggering neuroinflammation with consecutive neuronal loss, along with BDNF insufficiency [24].

A patient with Glut1DS, a novel *SLC2A1* mutation, and a hemangioma was described [71]. Glut1 expressed on endothelial cells is a selective marker of juvenile hemangioma, independent of mitotic activity [69]; nevertheless, the association may be coincidental [71]. Hemiplegic migraines due to vascular spasm were also described in Glut1DS [5].

### 8.2. Glut1 in Retina

In retina, Glut1-mediated transport occurs across the capillary endothelial cells of the inner blood–retinal barrier and the retinal pigmentary epithelium [72]. The rod photoreceptors secrete the rod-derived cone viability factor which binds basigin-1 expressed by photoreceptors, which in turn binds Glut1 [73]. Glut1DS may evolve with retinal changes and also with cataracts [5].

### 8.3. Glut 1 in Erythrocytes

Besides the brain tissue, Glut1 is highly expressed in erythrocytes, representing 5% of all of the membrane proteins [52]. Exercise-induced hemolytic anemia in Glut1DS is a consequence of cations draining through the defective Glut1 transporter [12]. Given the high expression of Glut1 on erythrocytes, red blood cell exchange transfusion—similar to sickle cell anemia—has been proposed, and is under study for the therapy of Glut1 patients (ClinicalTrials.gov Identifier NCT04137692, accessed on 20 April 2022).

### 8.4. Glut1 in Muscles

Glut1 is responsible for 30–40% of basal glucose uptake in skeletal muscle [74]. Patients with Glut1DS may have muscle hypotonia, and to some extent dysarthria and slurred speech may result from the involvement of the pharyngo-buccofacial system or the orofacial region muscles [29,75]. In the skeletal muscle, the mechanism regulating Glut-1 mediated glucose transport is more complex, as some Glut1 mutations decrease basal muscle transport much more than its surface expression [74,76].

### 8.5. Glut1 in Immune Cells

**Figure 4:**
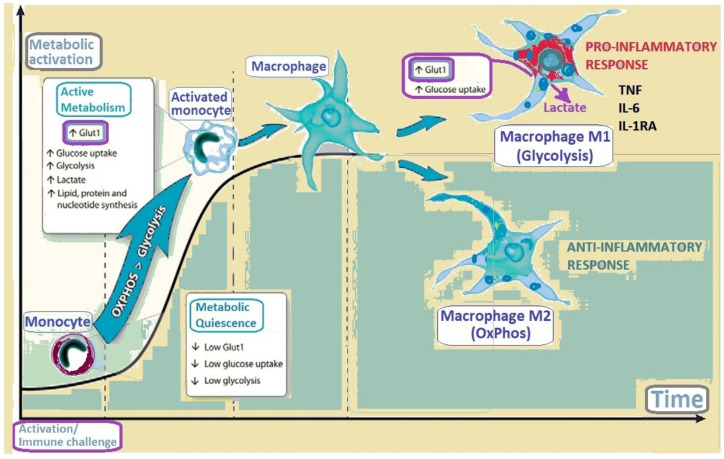
Immune challenge induces metabolic activation with increased *GLUT1* expression, glucose uptake and glycolysis. The transition of monocytes to macrophages is characterized by further increases in Glut1 expression and glycolysis. Naive (unactivated) monocytes are metabolically quiescent, with low basal metabolic activity and ATP derived primarily via oxidative phosphorylation (OxPhos). Classically activated macrophages (M1) induce aerobic glycolysis, resulting in lactate production and the increased production of inflammatory cytokines. Alternatively activated macrophages (M2) trigger a metabolic profile with OxPhos and an anti-inflammatory response. TNF: Tumor necrosis factor; IL-6: Interleukin 6; IL-1RA: interleukin-1 receptor antagonist.

At the interface and cross-talk of immunology and metabolism, the cellular metabolism regulates the fate, function and activation of the immune cells [77]. Metabolic adaptations and epigenetic reprogramming are involved in macrophage plasticity and phenotype change (Figure 4) [77,78,79,80,81]. Glut1 is also essential for the homeostasis of T and B lymphocytes [82,83]. Glut1 deficiency selectively impairs thymocyte and T effector functions [84]. In Glut1DS, the serum levels of antibodies are significantly lower, favoring the development of severe infections [45]. Furthermore, Glut1-deficient T cells are metabolically stressed, as their pAMPK level increases after activation, and cannot sustain activated mTORC1 signaling, while other signaling pathways such as Akt or ERK (extracellular regulated kinase) are generally unaffected [84,85].

## 9. Glut1 Inhibition in Other Settings

The relationship between glucose transport and metabolic pathways is worthwhile to explore for therapeutic purposes. Excessive glucose usage is related to several diseases, by linking immunological and metabolic pathways [68]. The glucose uptake should be efficient in cells with high turnover, such as immune cells and keratinocytes [68]. Warburg’s effect was described in tumors and in other high-energy-requiring cell processes [70]. The effects of Glut1 inhibition in health and pathology could provide clues for a better understanding of Glut1DS (see Table 3).

### 9.1. Glut1 in Keratinisation Disorders

Glucose transport in keratinocytes and wound- and inflammation-associated keratinocyte proliferation is mediated by Glut1 [68]. As Glut1 deficiency does not interfere with the epidermal development of function, Glut1 inhibition is a potential therapeutic strategy for psoriasis and other disorders of keratinization [68].

### 9.2. Glut1 in Eye Diseases

Targeting Glut1 with small molecules could alleviate retinitis pigmentosa by stimulating glucose uptake and preserving retinal cells [73]. Furthermore, Glut1 inhibition using small interfering RNAs reduces retinal glucose and improves diabetic retinopathy but may be detrimental to photoreceptors and retinal pigmentary epithelium [72,86].

### 9.3. Glut1 in Kidney Diseases

As Glut1 predominates in mesangial cells, as well as in podocytes, Glut1 activity alterations are deleterious in diabetic nephropathy, stimulating renal extracellular matrix production the most [87,88]. The *GLUT1* Enh2 genetic variation, located within a binding site for the insulin-responsive transcription factor, is associated with albuminuria, matrix expansion and glomerulosclerosis [88]. Podocytes enhance their glucose uptake but decrease their *GLUT1* expression when exposed to mechanical stress, suggesting alternative glucose transporters during stress/injury [87,89].

### 9.4. Glut1 in Placental Pathology

Glut1 expression changes during gestation, as Glut1 is expressed in placenta, syncytiotrophoblast, cytotrophoblast, and endothelial cells, and villous stroma [12]. Glut1 variations can result in significant transplacental glucose transport [8]. Glut1 is decreased in chronic hypoxia and in preeclampsia, but not in intrauterine growth restriction [12,90].

### 9.5. Glut1 in Heart Failure

Although Glut1 is the main glucose transporter in the heart, Glut-1 deficiency does not accelerate the progression from hypertrophy to heart failure, nor the pressure overload hypertrophy-induced mitochondrial dysfunction [91].

### 9.6. Glut 1 in Crystal-Induced Inflammation

In gout, the Glut1-mediated glucose uptake of the macrophages in the presence of uric acid is important for interleukin-1β production [81]. Glucose deprivation or therapy with a Glut1 inhibitor suppresses crystal-induced inflammation in gout and pseudogout, opening new therapeutic pathways [92].

### 9.7. Glut1 in Viral Infections

There are several viruses acting on the glucose transporters: Epstein-Barr, hepatitis B, HIV, Zika, rhinovirus or human cytomegalovirus (HCMV) [93]. Of note, Glut1 is a HTLV1 receptor molecule, and the infection downregulates the glucose uptake through Glut1 [94]. The HCMV early protein IE72 modifies the messenger RNA in the infected cells to downregulate *GLUT1* expression and to increase the more active *GLUT4* expression [93]. Due to the microcephaly accompanying in-utero Zika virus infections, an acquired Glut1 deficiency was suspected, but not confirmed [95]. In COVID-19, Glut1 and the ion transporter sodium proton exchanger 1 (NHE1) seem to be critically involved, and a low *GLUT1/NHE* RNA expression predicts disease severity in COVID-19 mostly in patients with cardiac complications [96].

### 9.8. Glut1 in Alzheimer’s Disease and Other Neurodegenerative Disorders

In Alzheimer’s disease (AD) there is cerebral glucose hypometabolism, partly due to reduced glucose transport at the BBB and across astrocytic and neuronal cell membranes [97]. In Alzheimer’s disease, vascular and non-vascular Glut1 and Glut3 are reduced in the hippocampus and cortex after beta-amyloid deposition, resulting in reduced glucose uptake [97]. Furthermore, Glut1 in AD is positively correlated with insulin signaling proteins [98]. In Alzheimer’s disease, the cause of Glut1 reduction is not known; it may be related to the low level of the regulator molecule HIF-1α, or to the direct effect of β-amyloid or tau on the glucose transporter gene expression [97]. Of interest, antidiabetic drugs like liraglutide may improve glucose brain transportation and reduce cognitive decline in AD [99].

As ^18^F-FDG-PET is used to image cerebral glucose consumption, it is also widely used in neurology for differential diagnosis with neurodegenerative diseases or encephalitis, in neurosurgery, or in psychiatry, mainly for atypical and/or pharmaco-resistant presentations [100,101]. Typical topographic patterns of glucose hypo-or hypermetabolism allow diagnosis and progression assessment for several diseases, such as dementia with Lewis bodies, frontotemporal lobar degeneration, and differential diagnosis between several neurodegenerative diseases including lateral amyotrophic sclerosis and Huntington’s disease, between Alzheimer’s disease and vascular dementia, or between parkinsonian syndromes associated with dementia, in neurooncology and others [100].

### 9.9. Glut1 in Cancers

**Figure 5:**
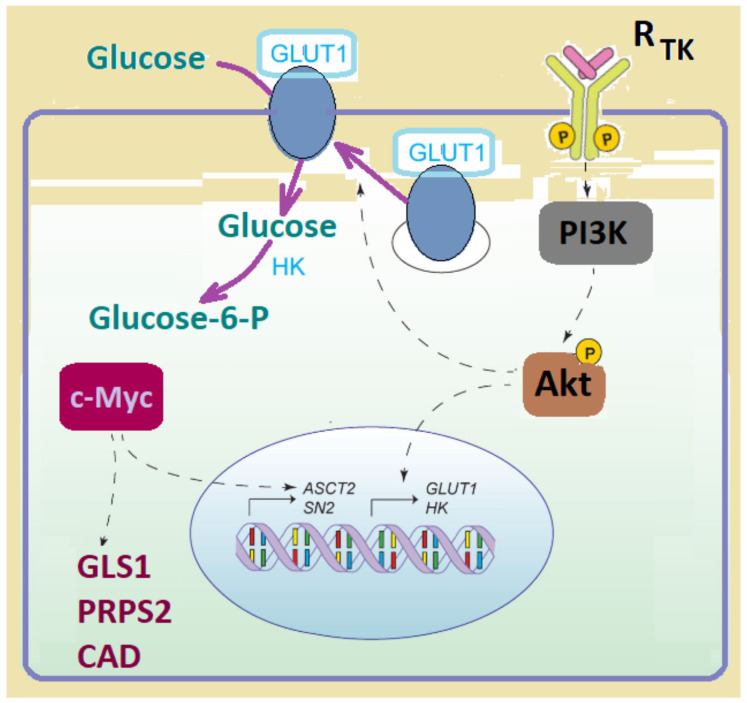
Aberrantly activated oncogenes deregulate the import of glucose through Glut1 into cancer cells. The solid purple arrows depict the metabolites and metabolic reactions. Dashed arrows depict regulatory effects of signal transduction components. Legend: Glut1, glucose transporter 1; HK, hexokinase; GLS1, glutaminase 1; PRPS2, phosphoribosyl pyrophosphate synthetase 2; CAD, carbamoyl-phosphate synthetase 2; RTK, receptor for tyrosine kinase; ASCT2/SN2, glutamine transporter gene.

As Glut1 seems to be preferentially used in cancers, Glut1DS patients may be naturally less prone to many types of cancer [102]. Glut1 is the predominant transporter in tumors, including hepatic, pancreatic, esophageal, brain, ovarian, cervical, renal, lung, cutaneous, colorectal and breast tumors, as well as head and neck cancers [102]. Neoplastic cells preferentially uptake the tracer in ^18^F-FDG-PET imaging [102]. Both *GLUT1* mRNA expression and Glut1 protein translocation from the endomembranes to the cell surface are promoted by PI3K/AKt signaling [103,104]. Targeting Glut1 with anti-Glut1 antibodies in breast cancer lines caused an up-to-75% reduction in proliferation [105]. Importantly, in animal models of mammary tumorigenesis, Glut1 loss prevented tumor formation without disrupting normal cell growth [102] (Figure 5).

**Table 3:** Glut1 in other settings: functional implications.

Cell/Tissue	Glut1	References
Vessels	Endothelial Glut1 is involved in vessel branching and migration in brain angiogenesis; Glut1 endothelial cell-specific haploinsufficiency was involved in triggering neuroinflammation	[24,68]
Retina	Glut1 depletion affects retinal angiogenesis and photoreceptor viability	[24]
Erythrocytes	Glut1 represents 5% of the erythrocyte membrane proteins In Glut1DS exercise may result in hemolytic anemia	[12,52]
Skin	Glut1 mediates glucose transport in keratinocytes, wound- and inflammation-associated keratinocyte proliferation	[68]
Muscle	Glut1 responds for 30–40% of skeletal muscle basal glucose uptake Glut1DS-associated muscle hypotonia may sometimes involve speech-associated muscles	[29,74,75]
Heart	Glut1—main glucose transporter in heart, but not critical for normal cardiac function	[91]
Placenta	Glut1 expressed in placenta, syncytiotrophoblast, cytotrophoblast, endothelial cells and villous stroma; Glut1 is decreased in chronic hypoxia and in preeclampsia, but not in intrauterine growth restriction	[8,90]
Kidneys	Glut1 expressed in glomerulus mainly in mesangial cells; Glut1 along with cytokines and growth factors favors diabetic glomerulosclerosis	[87,88]
Immune cells	Glut1 involved in macrophage plasticity and phenotype reprogramming in innate immune adaptations including in trained immunity; In gout interleukin-1 beta production depends on macrophage Glut1-mediated glucose uptake; Glut1 deficiency reduces T effector ability to induce inflammation, not affecting Tregs	[77,78,79,81,84]
Viral infections	Glut1 is a HTLV1 receptor molecule. The HCMV early protein IE72 downregulates *GLUT1* to increase *GLUT4* expression. In COVID19 Glut1 is critically involved, and a low Glut1/NPE-1 predicts COVID19 severity	[93,94,96]
Brain regions in Alzeimer’s disease	Glut1 and Glut3 are reduced in the hippocampus and cortex after β-amyloid deposition, resulting in reduced glucose uptake and metabolism	[97]
Cells in tumors	Glut1 is the predominant transporter in tumors, differentially required in different tumorigenesis stages. Blocking Glut1 inhibits tumorigenesis without disrupting normal cells.	[102,104]

Legend: HCMV, human cytomegalovirus; HTLV1, human T lymphotropic virus; NPE-1, sodium proton exchanger 1; Tregs, regulatory T cells.

## 10. Concluding Remarks and Future Directions

Glut1DS is considered to be a treatable inherited disease, but there are key issues regarding its diagnosis, treatment and long-term management. The main difficulty in diagnosing patients is the phenotypic heterogeneity related to the age and genetic complexity, underlining the need to increase physician awareness of this defect. The laboratory diagnostic for Glut1DS is the low CSF:blood glucose ratio (<0.45) after four–six hours of fasting, the molecular genetic test of the *SLC2A1* gene, or the red blood cell Glut1 surface expression test using flow cytometry analysis. The disease may be underdiagnosed. The KD still represents a standard choice in Glut1DS patients with favorable prognosis mostly involving the epileptic crises; with early treatment the patients continue to make progress and acquire mobility and speech. The standard therapy is still age-specific, based on ketogenic therapies that—by supplying ketones—are an alternative for brain fuel. The patients should avoid drugs that inhibit Glut1. There is still an overgrowing reluctance with respect to anticonvulsant administration which has been proven to have a poor response in epilepsy-related pathologies. Ongoing research to identify future interventions is focusing on small molecules designed to enhance Glut1 activity or expression, metabolic enhancement, and *SLC2A1* transfer strategies [5].

There are international consensus statements to facilitate the rapid diagnosis and multidisciplinary management of Glut1DS patients throughout their lives [5]. The evaluation of adult and pediatric Glut1DS patients is different [5]. Moreover, the first children diagnosed with Glut1DS are just coming of age. There are few data on pregnancy in this setting, with the dietetic therapy of the mother and infant having resulted in normal development in the early-treated neonate, emphasizing the need to identify and treat pre-symptomatic children [106]. The at-risk relatives of an affected child should also be tested as early as possible in order to minimize neurologic consequences. The need to identify pre-symptomatic individuals is an argument for newborn screening for Glut1DS [5,29].

Despite the improved prognosis, it is clear that there are unmet needs regarding the therapy of Glut1DS patients. Understanding the complex interactions of Glut1 with other tissues, its signaling function for brain angiogenesis and gliosis, and the complex regulation of glucose transportation and other metabolic pathways involving Glut1 in different tissues will hopefully also advance the therapy in this underdiagnosed disease [24]. The trafficking of Glut1 may be regulated by multiple pathways [74].

The upregulation of glucose transporters in other settings may shed a light on possible therapies for innate or acquired brain energy deficiencies. Short-term fasting upregulates glucose transporters in neurons and endothelial cells but not in astrocytes, as the neurons may be prioritized over astrocytes during fasting [107]. The study of supplements such as curcumin, which protects brain cells from apoptosis by upregulating Glut1 and Glut3, may be of interest [108]. Furthermore, other secondary deficits may be addressed, such as the metabolism of ascorbic acid, as Glut1 is also a prominent transporter of dehydroascorbic acid, the oxidized form of ascorbic acid [74]. In acquired forms of Glut1 deficiency such as Alzheimer’s disease, physical exercise may increase the Glut1 level [76].

A better understanding of the Glut1 functions in immune tissues will aid in characterizing the subtle immune deficiency in Glut1DS patients. Furthermore, the lack of significant impact on some tissues of Glut1 deficiency, despite the predominant expression of Glut1, implies coordinated compensatory mechanisms possibly involving other glucose transporters, which have to be unveiled for therapeutic purposes. This notwithstanding, recent research on the molecular and cellular impact of glucose deprivation helps us to define new therapeutic targets in Glut1DS and other syndromes with acquired glucose hypometabolism.

## Figures and Tables

**Table 1:** Major types of known glucose transporters [8,11,12,13,14].

Transporter	Main Substrate	Location	Main Properties	Type of Transport
Glut1	Glucose, galactose, mannose, glucosamine	RBC, kidney, colon, retina, placenta, myocardium, adipose tissue, brain, blood-brain barrier, blood-tissue barrier, many fetal tissues	Glucose uptake in most of cells, expression is age-related	Passive transport, sodium-independent transporters
Glut2	Glucose, galactose, fructose, mannose, glucosamine	Serosal surface of intestinal cells, liver, beta cells of pancreas, kidney	Low affinity; glucose uptake in liver; glucose sensor in pancreatic beta cells	
Glut3	Glucose, galactose, mannose, xylose	Brain (neurons membrane), testis	High affinity; transports glucose into brain cells	
Glut4	Glucose, glucosamine	Skeletal and cardiac muscle, adipose tissue [white and brown]	Insulin mediated glucose uptake, expression is age-related	
Glut5	Fructose	Small intestine, kidney	Poor ability to transport glucose; is mainly a fructose transporter	
Glut6	Glucose	Spleen, leucocytes, brain	Glucose transport	
Glut7	Glucose, fructose	Liver endoplasmic reticulum, small intestine, colon, testis, prostate	Glucose transport from ER to cytoplasm	
Glut8	Glucose, fructose, galactose	Testis, brain, blastocyst, adrenal gland, liver, spleen, muscle, brown adipose tissue, lung [intracellular]	Glucose/(Fructose) transport	
Glut9	Urate (glucose, fructose)	Liver, kidney, small intestine, placenta, lung, leukocytes	Glucose/Fructose transport, not galactose	
Glut10	Glucose, galactose	Heart, lung, brain, liver, skeletal muscle, pancreas, placenta, kidney, mitochondria of smooth muscle cells	Facilitates DHAA, import into mitochondria of smooth muscle cells and insulin stimulated adipocytes; protects cells against oxidative stress, connects mitochondrial function to TGF-β signaling	
Glut11	Glucose, fructose	Heart, kidney, skeletal muscle, adipose tissue and pancreas	The 3 Glut11 variants are differentially expressed; primary physiological substrates have not been definitively identified	
Glut12	Glucose; also transports α-methyl-D-glucopyranoside	Heart, renal tubules, digestive tube epithelium, prostate, adipose tissue, liver, skeletal muscle, placenta, thyroid, adrenal and pituitary glands	The role in glucose homeostasis under normal or pathophysiological conditions is not fully understood; but insulin has been reported to acutely stimulate the translocation of Glut12 from intracellular membrane compartments to the plasma membrane in human skeletal muscle	
Glut13 (also called HMIT]	Myo- inositol	Muscle, thyroid, adrenal and pituitary glands, kidney, white and brown adipose tissue; brain (both in neurons and glial cells): highly expressed in the hippocampus, hypothalamus, cerebellum, brainstem	In neurons is present in intracellular vesicles involved in increasing myo-inositol uptake. Possible role in regulating processes such as membrane recycling, growth cone dynamics and synaptic vesicle exocytosis (requiring high levels of myo-inositol or its derivatives).	
Glut14		Testis	The role is not fully understood; his gene (*SLC2A14*) shares 95% sequence identity with the Glut3 gene and, therefore, appears to be encoded by a gene duplication.	
SGLT (sodium-dependent transporters]		SGLT1 in intestine, in kidney	Co-transport; from lumen into cells.	Active transport
		SGLT2 in kidney		
SWEETs mediate mainly the efflux of glucose in humans and are ubiquitous in human body		They have the highest expression in the oviduct, epididymis and intestine; also are localized in pancreatic beta cells. Further studies are required to discover SWEET physiology in humans.	SWEETs may function as uniporters, although this hypothesis remains unproven. Have the ability to transport various mono- and disaccharides, the ability to mediate both cellular uptake and efflux, and have typically low affinities for sugars.	Passive transport, sodium-independent transporters

Legend: RBC, red blood cells; HMIT, proton-driven myo-inositol co-transporter; ER, endoplasmic reticulum, DHAA, dehydroascorbic acid; SGLT, sodium-glucose linked transporter (co-transporters); TGF-β, transforming growth factor-β.

**Table 2:** Recommended treatments in cases with the epilepsy-associated phenotype of Glut1DS; diet treatments and antiepileptic drugs (AED) to avoid.

Diet/Treatment	AED Indicated	Drugs Not Recommended in Association with KD	References
Ketogenic diet (KD)	Acetazolamide	Valproate	[5,12,56]
Modified Atkins Diet	Topiramate	Zonisamide	[56]
Medium chain Triglycerides	Zonisamide	Acetazolamide	[5,12,56]
Low glycemic index treatment	Phenytoin	Topiramate	[5,56]
Triheptanoin	Carbamazepine	-	[56,64]
α-lipoic acid (under investigation)	-	-	[5,12,56]

## Data Availability

Not applicable.
